# Distinct activation of the sympathetic adreno-medullar system and hypothalamus pituitary adrenal axis following the caloric vestibular test in healthy subjects

**DOI:** 10.1371/journal.pone.0193963

**Published:** 2018-03-06

**Authors:** Sebastian Cozma, Cristina Mihaela Ghiciuc, Lisandra Damian, Vittorio Pasquali, Angelo Saponaro, Elena Catalina Lupusoru, Francesca Romana Patacchioli, Lucia Corina Dima-Cozma

**Affiliations:** 1 Department of Otorhinolaryngology, Rehabilitation Hospital, School of Medicine, University of Medicine and Pharmacy “Grigore T. Popa”, Iasi, Romania; 2 Department of Pharmacology, School of Medicine, University of Medicine and Pharmacy “Grigore T. Popa”, Iasi, Romania; 3 Department of Psychology, Sapienza University of Rome, Rome, Italy; 4 Department of Physiology and Pharmacology “V. Erspamer”, Sapienza University of Rome, Rome, Italy; 5 Department of Internal Medicine, Rehabilitation Hospital, School of Medicine, University of Medicine and Pharmacy “Grigore T. Popa”, Iasi, Romania; UCSD, UNITED STATES

## Abstract

**Introduction:**

The vestibular acute stress induces reversible alert-like reactions that involve the sympathetic adrenal-medullar system and hypothalamic-pituitary-adrenal axis responses. The present study aimed to evaluate salivary α-amylase and salivary cortisol production in relation with cardiovascular reactivity induced by acute stress in healthy subjects.

**Material and methods:**

Forty-eight young healthy male volunteers were examined under basal conditions and at various times after reaching the maximal nystagmic reaction following air caloric vestibular test. Heart rate, systolic blood pressure, diastolic blood pressure and mean arterial pressure were recorded at the same time as measurement of the salivary α-amylase and salivary cortisol. At the end of the caloric vestibular test session, perceived stress scale questionnaires were administered to measure the self-perceived stress impact induced by the task, and individual scores were compared with those measured on the enrollment day.

**Results:**

Following caloric vestibular test-evoked vertigo, salivary α-amylase and cortisol showed distinct trends in their production after acute stress: Student’s t-test was used to compare the α-amylase vs cortisol slopes of the respective interpolated regression lines, and the difference was significant (t = -3.283; p<0.001); an increase in salivary cortisol production corresponded with a decrease in the salivary α-amylase concentration. In addition, salivary biomarker modifications were associated with consistent changes in the heart rate, systolic blood pressure and mean arterial pressure.

**Conclusions:**

Using the air caloric vestibular test task as a stressor, the present study demonstrated a connection between the acute hormonal stress response to vestibular stimulation and cardiovascular output. However, further research is needed before we can define the potential importance of the consistent cardiovascular activity changes evoked by vestibular stimulation and the possible functional consequences for cardiovascular regulation and orthostatic tolerance in humans.

## Introduction

Several studies have examined the autonomic effects of vestibular stimulation, suggesting that vestibular imbalance is a stressful condition in itself [1] and caloric vestibular test (CVT)-evoked vertigo may generate some uneasiness in patients, eliciting an acute and reversible alert-like reaction that involves the sympathetic adrenal-medullar (SAM) system and hypothalamic-pituitary-adrenal (HPA) axis responses. Measurements of salivary α-Amylase (α-Amy) and cortisol as neuroendocrine subclinical indicators of the SAM system and HPA axis activities are increasingly used for not invasive monitoring of the response of the human body to stressful challenges under different physio-pathological conditions [2–11].

We hypothesized that the air CVT-evoked vertigo challenge alters salivary α-Amylase and cortisol production. The study subjects were investigated under the basal condition and at various times for 60 minutes after reaching the time of maximal nystagmic reaction following caloric irrigation. Forty-eight healthy male volunteers (median age 24 years, range 21–30 years) entered the study and were also examined through standardized self-reported questionnaires aimed at identifying their mental health and subjective stress perception levels.

Because the available literature does not provide clear evidence of the temporal sequence of the contribution of the vestibular system to the sympathetic cardiovascular regulation during the acute stress response [12, 13], the present study was conducted with a twofold purpose: to simultaneously evaluate cardiovascular activity during the aversive acute task of the air CVT stimulation by measuring several cardiovascular parameters in the study population, including heart rate (HR), R to R wave interval (RR), systolic blood pressure (SBP), diastolic blood pressure (DBP) and mean arterial pressure (MAP), which were recorded while measuring the salivary biomarkers.

## Materials and methods

### Study population

This prospective study was formally approved by the Ethics Committee of the Iaşi “Grigore T. Popa” University of Medicine and Pharmacy (protocol n° 192/January 7, 2014).

In the a priori sample size calculation, we estimated that at least 23 subjects were required to detect a mean absolute difference of approximately 25% for the expected changes in salivary cortisol and/or α-Amy concentrations between the values measured before and after the air CVT-evoked vertigo challenge, with α of 0.05, β = 0.2 and statistical power of 80%. A total of 62 moderately active (moderate physical activity not exceeding > 2 hours and 30 minutes spread throughout the week), healthy Caucasian males were recruited among students attending the Iasi Universities. For all subjects, the medical examinations, main hematological and blood chemistry parameters, and electrocardiograms were within normal ranges; metabolic, cardiovascular, endocrine and other chronic diseases were considered as the exclusion criteria. After their initial enrollment in the study, 12 subjects were excluded because they incorrectly collected the salivary samples, and 2 were excluded because they occasionally took drugs that were not allowed in the three days preceding the experimental procedure.

None of the study participants had received any anti-inflammatory or immunosuppressive drug in the previous 6 months, nor had they received any vasoactive drugs that could have influenced α-Amy or cortisol secretion (e.g., antihypertensive, antidepressants, thyroid tropic drugs). None of the participants had previously undergone the CVT task or had a clinical presentation compatible with audiological and vestibular pathology. During the three days preceding the air CVT task, all participants were asked to avoid alcohol and chocolate and to follow a dietary schedule ensuring a daily total caloric intake of ~ 2600 kcal/day, which is close to the recommended dietary allowance (RDA) for this population of moderately active young healthy male subjects [14]; moreover, the study participants were asked to maintain a complete resting state, without engaging in any intense physical activity or receiving any significant psychological input.

### Experimental protocol

Written informed consent was obtained from all participants during a preliminary informative meeting, which was followed by 3 subsequent experimental sessions. In the first session, they underwent baseline cardiac evaluations (with electrocardiogram) and an ENT physical examination that included otoscopy, tympanometry, audiometry and clinical balance tests to check the anatomical integrity and functionality of the audio-vestibular system. The routine laboratory tests (complete blood count, lipids and lipoproteins, fasting blood glucose, hepatic, renal, and thyroid profiles) were all within normal ranges.

In the second session, we performed a psychometric screening of the study population to exclude any psychopathologies. Upon arrival, each participant was seated in a comfortable chair and given a full explanation of the tests about to be undertaken. Then, the participant completed the Hamilton Rating Scale for Depression (HDS) [15] and the Hamilton Anxiety Rating Scale (HAS) [16]. Furthermore, the Perceived Stress Scale (PSS) [17] was administered to measure individual subjective stress perception scores in the study population. The items in the PSS were designed to assess how unpredictable, uncontrollable and overwhelming the subjects found their lives. The participants responded on a 5-point scale ranging from 0 (”never”) to 4 (”very often”). The responses to the items represented a psychological stress score, with higher scores indicating greater psychological stress [17]. Stress level perceptions were also indicated by the Hassles questionnaire scores, which were recorded after the air CVT challenge and compared with those reported during the enrollment session [18].

At the end of the second session, all subjects were taught how to collect their saliva at home using the Salivette sampling device (Sarstedt, Germany), and they were asked to avoid food, coffee, teeth brushing and any physical exercise for at least 30 min before each saliva collection [11].

Home diurnal saliva collection was scheduled at 8:00 (at least two hours after awakening), before lunch (at 12:00) and before dinner (at 20:00). The day after the home saliva collection, the subjects attended the third experimental session (always in the morning between 9:00 and 12:00), when the air CVT was performed. Air CVT was conducted according to the British Society of Audiology guidelines 2010 [19] and Ganança and coworkers [20]. During the caloric test, the subjects were positioned in the supine position with their heads and backs inclined at 30° to the horizontal. Subsequently, the participants were fitted with infrared video Frenzel Goggles (Difra, Eupen, Belgium) connected to a video-nystagmoscopy monitor and equipped with a screen chronometer to monitor the appearance, direction and duration of ocular nystagmus. In the present protocol, the air CVT stimulation was performed by irrigating the external auditory canal of the right ear with a flow of 50°C warm air (Air Fx Caloric stimulator, Interacoustics, Denmark) for 60 seconds [21]. In a patient with normal horizontal semicircular canal function, this process will result in approximately two minutes of horizontal nystagmus in the same direction of the stimulated labyrinth. We performed the procedure for one single ear to avoid any predictability in the subjective vestibular sensation for the second ear, considering that all volunteers were selected after excluding any optic or audio-vestibular pathology, and they had never experienced the CVT before. After air irrigation and observation of the nystagmus, the patients were asked to rate the level of their symptoms of dizziness on a Likert scale ranging from 1 to 10, with 1 indicating no dizziness and 10 being the most intense sensation of dizziness [22]. The duration of the induced nystagmus was measured by the audiologist on the screen chronometer.

Ten minutes before the air CVT task, the subjects were attached to a 12 channel Holter (BTL-08 CardioPoint-Holter, USA) for continuous monitoring of the electrocardiogram. The SBP and DBP were measured using a semi-automatic sphygmomanometer (M3; Omron, Matsusaka Co. LTD, Japan). MAP was calculated as (2DBP + SBP)/3 [23]. Salivary samples were collected 5 minutes before the CVT task (under the basal experimental condition) and 1, 4, 7, 10, 15, 30, 45 and 60 min thereafter, at the same time as the cardiovascular measurements. At all these experimental session time points, the SBP and DBP were sequentially determined, while the corresponding HR and R to R wave interval were selected from the Holter recording.

At the end of the CVT task session, PSS questionnaires were administered to measure the self-perceived stress impact induced by the task, and individual scores were compared with those measured on the enrollment day.

### Saliva collection and salivary biomarker assay

Saliva was collected using the Salivette sampling device (Sarstedt, Germany), which allows for quick and hygienic saliva recovery from a polyester swab through centrifugation at 3,000 rpm for 15 min [5]. Salivary samples were immediately frozen at –20°C until the analysis. α-Amy and cortisol assays were performed, as previously described [5], using commercially available assay kits (Diametra, Italy).

### Data analysis and statistic

Unless otherwise specified, the data are reported as the mean ± SD. Statistical analyses and graphics were performed using the Sigma Plot 11 (SxST.it, Italy) and Statistica 6.0 (Statsoft Inc, USA) software programs.

A repeated measures ANOVA test was performed to reveal the diurnal variation of the α-Amy and cortisol secretions and the effect of air CVT on the salivary cortisol and α-Amy concentrations, as well as on SBP, DBP, HR and RR, which were simultaneously recorded with the salivary collections during the air CVT task. Post hoc analyses, using Tukey’s multiple comparison test, were conducted to reveal subgroup differences. To evaluate the trend of each salivary biomarker production in the 15 min following the air CVT stress-induced response, Pearson’s coefficient for salivary biomarker concentration against time was determined; when the resulting r value was significant, an equation that described the interpolated regression line was derived, and Student “t” test for slope comparisons was applied [24, 25].

The statistical significance was set at p<0.05.

## Results

### Characteristics of the study population

Somatic and psychometric characteristics of the study population at the time of enrollment are shown in Table 1. Body mass index (BMI), which compares height to weight, indicated that the study population was within the normal range (normal values are 18.5–24.9 kg/m^2^), and waist circumference, an indicator of cardiometabolic risk, showed that the study population was below 94 cm, the lower risk level for men [26]. In addition, Table 1 shows that the basal cardiovascular parameters (SBP, DBP and HR) were within the normal range in the study population. Table 1 also shows that the subjects’ HDS (<7) and HAS (<20) scores indicated that the study population demonstrated no signs of depression or anxiety.

**Table 1:** Somatic and psychometric variables of the study population (n = 48).

Age (years)	25 ± 2
BMI (kg/m^2^)	23.6 ± 2.9
Waist circumference (cm)	86 ± 7
Educational level (years)	17 ± 2
SBP (mmHg)	121 ± 12
DBP (mmHg)	77 ± 9
HR (beats/min)	72 ± 9
HDS	3.5 ± 2.1
HAS	3.0 ± 2.1

Data are expressed as the mean ± SD.

List of abbreviations: body mass index, BMI; Hamilton Anxiety Score, HAS; Hamilton Depression Score, HDS; heart rate, HR; systolic blood pressure, SBP; diastolic blood pressure, DBP.

**Fig 1:**
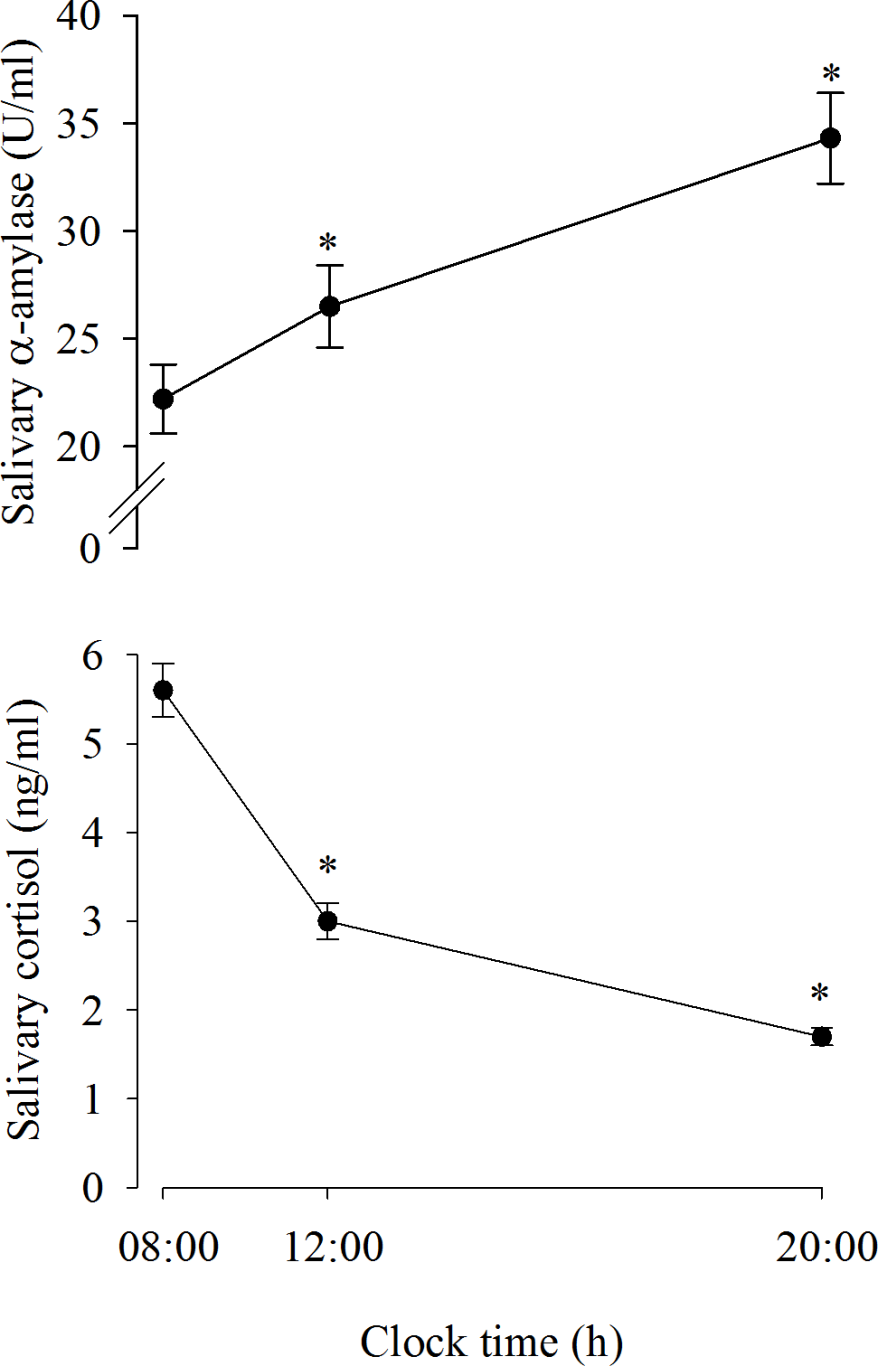
Diurnal trajectories of salivary α-amylase and salivary cortisol during a rest day. Data are expressed as the mean±SE. *: p<0.001 vs 08:00.

Fig 1 (upper part) shows the mean levels (±SE) of α-Amy measured in the study population at 08:00 h (22.2±1.6 U/ml), 12:00 h (26.5±1.9 U/ml) and 20:00 h (34.3±2.1 U/ml) during a resting day. The One-way repeated measures ANOVA revealed significant effects for the factor time F (2,143) = 19.809, p<0.001. The post hoc Fisher LSD method for multiple comparisons showed that the α-Amy diurnal pattern, with levels measured at 08:00 h, was significantly lower than those measured at 12:00 h (p<0.001) and at 20:00 h (p<0.001). Fig 1 (lower part) shows the mean levels (±SE) of salivary cortisol measured in the study population during a resting day. The one-way repeated measures ANOVA revealed significant effects for the factor time F (2,143) = 135.843, p<0.001. The post hoc Fisher LSD method for multiple comparisons showed that the salivary cortisol concentrations measured at 12:00 h (3.0±0.2 ng/ml) and 20:00 h (1.70±0.1 ng/ml) were significantly lower (p<0.001) than those measured at 08:00 h (5.60±0.30 ng/ml).

### Air CVT-evoked nystagmus, dizziness and stress perception in the study population

The latency (41±2 seconds) and the duration of the nystagmus (127±28 seconds) were measured in the participants as an objective sign of the induced vertigo. As a subjective uneasiness symptomatology induced by the air CVT challenge, a quite intense sensation of dizziness (7.5±2, on a 10-point Likert scale) was reported by the study participants.

Table 2 shows the reported self-rated measurements of stress perception registered at the end of the air CVT task compared with those measured on the enrollment day. For the PSS questionnaire, the participants demonstrated a score (29±3) indicated that they perceived a low level of stress during the enrollment session. According to the PSS questionnaire rating scale, the data indicated that at the end of the air CVT task, the subjects showed signs of a medium level of stress perception, with individual scores significantly higher than those perceived by the subjects during the enrollment session (Wilcoxon signed-rank test, z = 6.043, p<0.001). A medium stress level perception was also indicated by the Hassles questionnaire scores, which were recorded after the air CVT challenge, compared to the low stress level perceived by the participants during the enrollment session (Wilcoxon signed-rank test, z = 6.046, p<0.001).

**Table 2:** Self-reported stress perception in the study population (n = 48).

	Rest day	CVT day
PSS scores	29 ± 3	35 ± 4*
Hassles scores	73 ± 7	87 ± 12*

Data are expressed as the mean ± SD.

*: p < .001 versus value at rest day.

List of abbreviations: Perceived Stress Scale (PSS)

### Air CVT-evoked change in α-Amy and cortisol production in the study population

**Fig 2:**
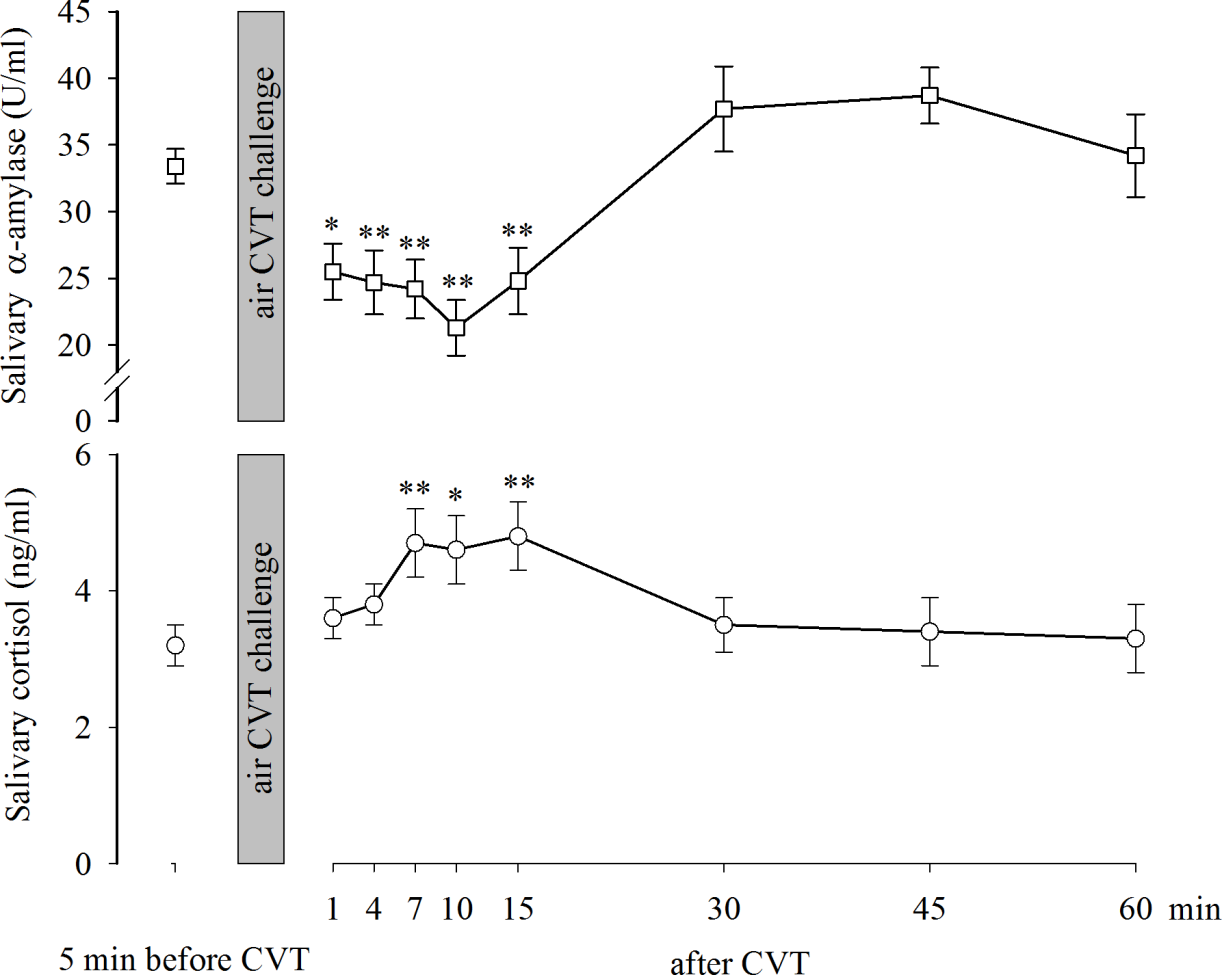
Diurnal trajectories of salivary α-amylase and cortisol before and after CVT stimulation. Data are expressed as the mean±SE. *,**: p<0.01 and p<0.001, respectively, vs before the CVT.

Fig 2 (upper part) shows the mean (±SE) α-Amy levels measured in the study population before the air CVT challenge (33.4±1.3 U/ml) and at various times thereafter (after 1 min: 25.5±2.1 U/ml; after 4 min: 24.7±2.4 U/ml; after 7 min: 24.2±2.2 U/ml; after 10 min: 21.3±2.1 U/ml; after 15 min: 24.8±2.5 U/ml; after 30 min: 37.7±3.2 U/ml; after 45 min: 38.7±2.1 U/ml, and after 60 min: 34.2±3.1 U/ml). The one-way repeated measures ANOVA revealed significant effects at various times after the air CVT challenge F(8,431) = 13.584, p<0.001. The Tukey test for multiple post hoc comparisons showed that the α-Amy concentrations measured 1 min (p<0.01), 4 min (p<0.001), 7 min (p<0.001), 10 min (p<0.001) and 15 min (p<0.001) after the end of the air CVT task were significantly lower than those measured before the task. Fig 2 (lower part) shows the mean (±SE) salivary cortisol measured in the study population before the air CVT challenge (3.2±0.3 ng/ml) and at various times thereafter (after 1 min: 3.6±0.3 ng/ml; after 4 min: 3.8±0.3 ng/ml; after 7 min: 4.7±0.5 ng/ml; after 10 min: 4.6±0.5 ng/ml; after 15 min: 4.8±0.5 ng/ml; after 30 min: 3.5±0.4 ng/ml; after 45 min: 3.4±0.5 ng/ml, and after 60 min: 3.3±0.5 ng/ml). The one-way repeated measures ANOVA revealed significant effects at different times after the air CVT challenge F(8,431) = 4.432, p<0.001. The post hoc Tukey test for multiple comparisons showed that the salivary cortisol concentration measured 7 min (p<0.001), 10 min (p<0.01) and 15 min (p<0.001) after the end of the air CVT task were significantly higher than the levels measured before the task.

### Correlation between air CVT–evoked α-Amy and cortisol production in the study population

The effect of the stressor was significant during the first 15 min following the air CVT stimulation. Therefore, to evaluate the trend of each salivary biomarker production during this period, Pearson’s coefficient for salivary biomarker concentration against time was determined. Because the resulting r values were significant, an equation describing the interpolated regression line was derived. The results (scatterplots are not shown) showed the following regression lines for α-Amy (N = 288): yα-Amy = 27.6618–0.4195x (r = -0.2023, p = 0.00055). The following regression lines were demonstrated for salivary cortisol (N = 288): yCortisol = 3.7387 + 0.0774x (r = 0.198, p = 0.000715). The t-tests for the slope of the α-Amy line vs the cortisol line (slope α-Amy = -0.4195; slope Cortisol = 0.0774) yielded a p<0.001 statistically significant difference, with t = -3.283 (DF = 575), indicating that parallelism was not present among the lines. Thus, the results showed that while α-Amy production decreased after stress, salivary cortisol production increased.

### Air CVT-evoked cardiovascular activity changes in the study population

**Fig 3:**
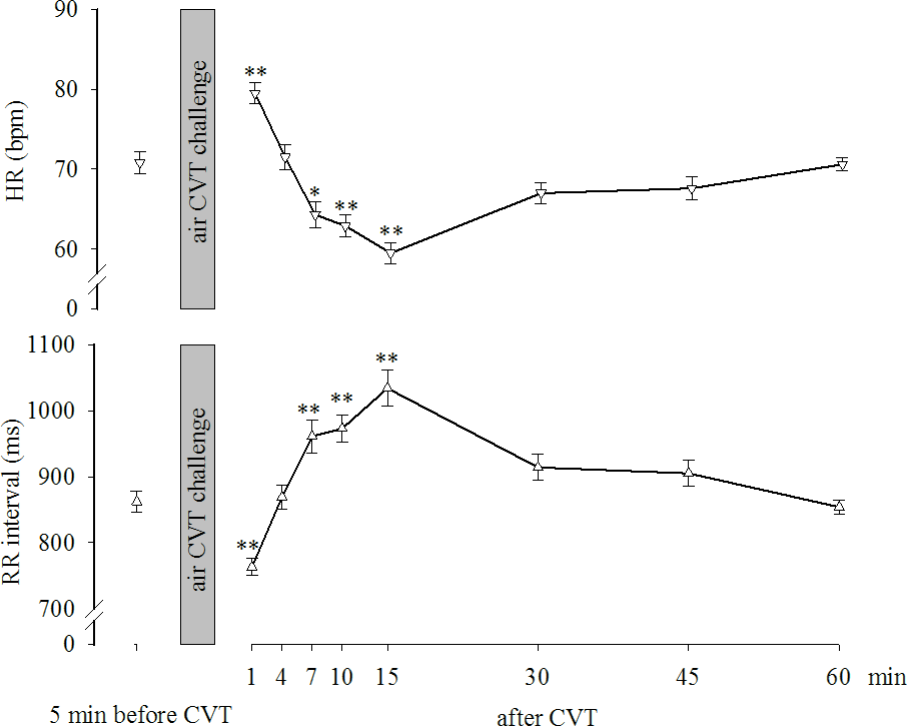
HR and RR intervals before and after the CVT stimulation. Data are expressed as the mean±SE. *, **: p<0.01 and p<0.001, respectively, vs before the CVT.

Fig 3 (upper part) shows the HR in the study population before (71±1.4 bpm) and at various times after the air CVT task (1 min after: 79±1.3 bpm; after 4 min: 71±1.5 bpm; after 7 min: 64±1.6 bpm; after 10 min: 63±1.4 bpm; after 15 min: 60±1.3 bpm; after 30 min: 67±1.3 bpm; after 45 min: 68±1.4 bpm and after 60 min: 71±0.8 bpm). The one-way repeated measures ANOVA revealed that significant effects were induced by the air CVT challenge during the time thereafter F(8,431) = 27.027, p<0.001. The post hoc Tukey Test for multiple comparisons showed that the HR measured 1 min after the end of the air CVT challenge was significantly (p<0.001) higher than that measured before the task. In contrast, the HRs measured 7 min (p<0.01), 10 min (p<0.001) and 15 min (p<0.001) after the end of the air CVT task were significantly lower than those before the test. Fig 3 (lower part) shows the RR interval in the study population before (862±16 ms) and at various times after the air CVT task (after 1 min: 763±13 ms; after 4 min: 869±18 ms; after 7 min: 961±25 ms; after 10 min: 973±21 ms; after 15 min: 1034±27 ms; after 30 min: 914±20 ms; after 45 min: 905±20 ms, and after 60 min: 854±9 ms). The one-way repeated measures ANOVA revealed significant effects induced by the air CVT challenge during the time thereafter F(8,431) = 26.262, p<0.001. The post hoc Tukey test for multiple comparisons showed that the RR interval measured 1 min after the air CVT challenge was significantly (p<0.001) lower than that measured before the task. In contrast, the RR intervals measured 7 min, 10 min and 15 min after the end of the air CVT task were significantly (p<0.001) higher than those measured before the test.

**Fig 4:**
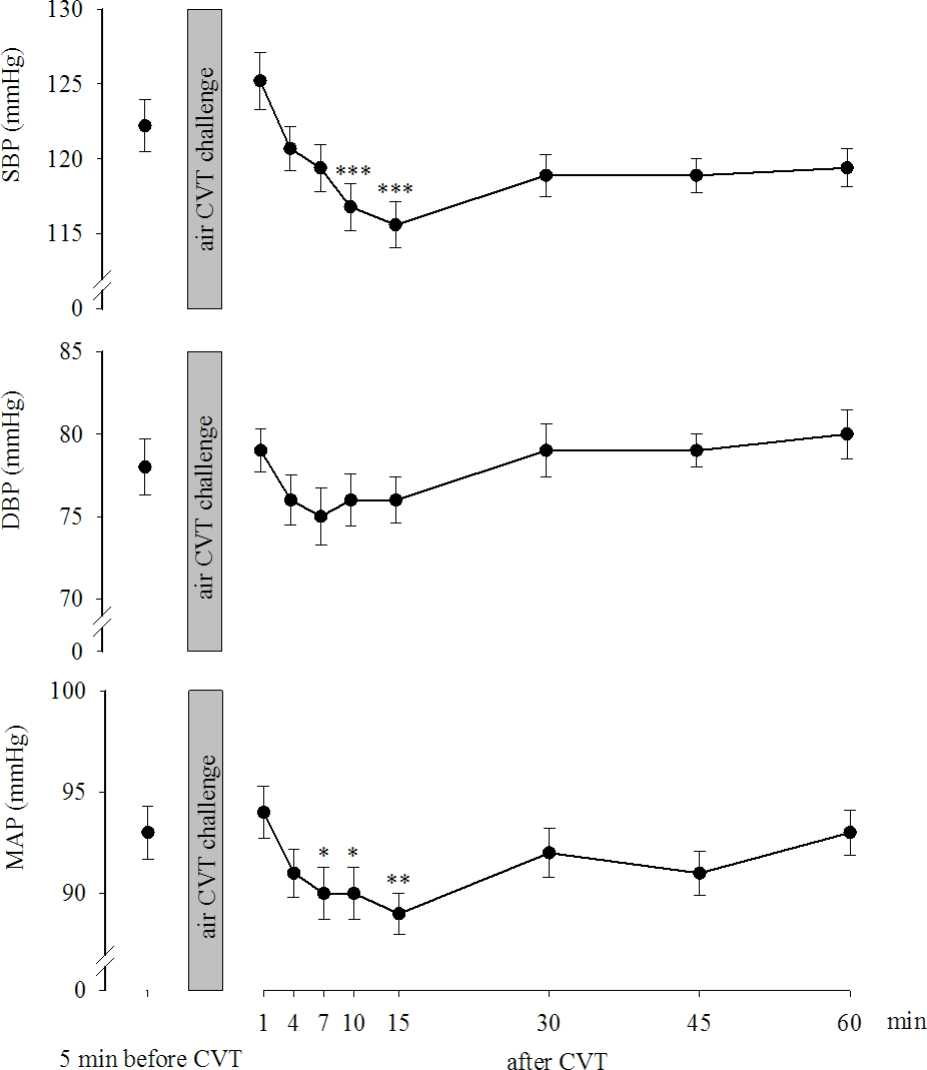
SBP, DBP and MAP before and after the CVT stimulation. Data are expressed as the mean±SE. *,**,***: p<0.05, p<0.01 and p<0.001, respectively, vs before the CVT.

Fig 4 (upper part) shows the SBP in the study population before the air CVT challenge (122±2.2 mmHg) and at various times thereafter (after 1 min: 125±2.5 mmHg; after 4 min: 121±1.9 mmHg; after 7 min: 119±2.0 mmHg; after 10 min: 117±2.0 mmHg; after 15 min: 116±2.0 mmHg; after 30 min: 119±1.5 mmHg; after 45 min: 119±1.4 mmHg and after 60 min: 119±1.6 mmHg. The one-way repeated measures ANOVA revealed significant effects induced by the air CVT challenge during the time thereafter F(8,431) = 11.179, p<0.001. The post hoc Tukey test for multiple comparisons showed that the SBPs measured 10 min (p<0.001) and 15 min (p<0.001) after the end of the air CVT task were significantly lower than those measured before the task. Fig 4 (middle part) shows the DBP levels in the enrolled subjects before the air CVT challenge (78±1.7 mmHg) and at various times thereafter (after 1 min: 79±1.3 mmHg; after 4 min: 76±1.5 mmHg; after 7 min: 75±1.7 mmHg; after 10 min: 76±1.6 mmHg; after 15 min: 76±1.4 mmHg; after 30 min: 79±1.6 mmHg; after 45 min: 79±1.8 mmHg, and after 60 min: 80±1.5 mmHg). The one-way repeated measures ANOVA revealed significant effects induced by the air CVT challenge during the time thereafter F(8,431) = 5.370, p<0.001. The post hoc Tukey test for multiple comparisons showed no significant effects on DBP compared with those measured before the task in the study population. Fig 4 (lower part) shows the MAP calculated in the enrolled subjects before the air CVT challenge (93±1.7 mmHg) and at various times thereafter (after 1 min: 94±1.6 mmHg; after 4 min: 91±1.5 mmHg; after 7 min: 90±1.7 mmHg; after 10 min: 90±1.6 mmHg; after 15 min: 89±1.4 mmHg; after 30 min: 92±1.3 mmHg; after 45 min: 92±1.2 mmHg, and after 60 min: 93±1.2 mmHg). The one-way repeated measures ANOVA revealed significant effects induced by the air CVT challenge during the time thereafter F(8,431) = 8.069, with p<0.001. The post hoc Tukey test for multiple comparisons showed that the MAPs measured 7 min (p<0.05), 10 min (p<0.05) and 15 min (p<0.01) after the end of the air CVT task were significantly lower than those measured before the task.

## Discussion

We hypothesized that the air CVT-evoked vertigo challenge provokes alterations in α-Amy and cortisol production. Here, we report that the cortisol variation generated by the exposure to the air CVT-evoked vertigo in healthy young subjects was consistently associated with an alarm reaction in the HPA axis and a reduction in sympathetic activity, as shown by the reduced α-Amy secretion. Thus, the two systems act distinctly in response to stress, which has been previously shown in asthmatic children [27] and in maltreated youth [28]. Furthermore, the transitory stress-induced modifications of the salivary biomarkers were associated with consistent transient changes in HR, SBP and MAP during the time following the vestibular stimulation.

In the present study, we demonstrated that the air CVT, a useful medical examination procedure that assesses the horizontal semicircular canal function of each labyrinth and is severe enough to provoke nystagmus and dizziness, is a powerful activator of the HPA axis, a finding that aligns with previous observations made by Dagilas and coworkers [29], who demonstrated a large increase in cortisol levels after the air CVT.

Under basal non-challenging conditions, the study population showed the expected significant diurnal fluctuation of salivary cortisol levels, with the highest concentrations measured in the morning and the lowest concentrations measured in the evening. Concomitantly, in the same subjects, a significant increase in α-Amy was detected in the evening compared to the morning [5]. Thus, salivary cortisol and α-Amy have opposite diurnal fluctuation patterns: as evening approaches, cortisol concentrations decrease, and α-Amy activity increases [5, 8]. Overall, salivary cortisol levels reflect the activity of the HPA axis, whereas α-Amy is a marker of the sympathetic activity. Using air CVT task as a stressor, the present study indicates a connection between the acute hormonal stress response to vestibular stimulation and cardiovascular output. Given the potential interaction between the vestibular and autonomic pathways, this finding could be explained by the reduction in the SAM system activity following vestibular stimulation [30]. Several clinical observations and animal studies have suggested a link between vestibular and autonomic systems [12, 29, 31, 32]. The nature of these interactions, however, is complex and has not been fully defined. In fact, rather than a sympathetic inhibition, vestibular stimulation has consistently been shown to increase the sympathetic outflow in cardiac and splanchnic vascular beds in most experimental models [1, 12] or even to induce inconsistent changes in HR and BP, despite substantial symptoms of motion sickness in humans [31].

Our study aligns with other previously published papers that have reported that stress-dependent variations in α-Amy occurred in conjunction with cardiovascular parameter responses, reflecting autonomic function [9, 33–36]. We reported a significant decrease in HR, SBP and MAP during the first 15 minutes of observation following the air-CVT challenge, as well as an increase in the pretest values thereafter, which aligned with the α-Amy response. Chatterton and coworkers showed rapid stress-induced increases in α-Amy and heart rate. Moreover, they reported that α-Amy was correlated with plasma norepinephrine concentrations after a physical exercise challenge [33]. Nater and coworkers [34] reported a positive correlation between heart rate variability and α-Amy variation after the Trier Social Stress Test, showing no correlation between plasma catecholamine and α-Amy. Furthermore, psychological stress-induced changes in α-Amy correlated with SBP and plasma catecholamine levels [35]. More recent investigations have described analogous trajectories of HR and α-Amy levels over and after the effort test challenge [9, 36].

The protocol of the present study provided a multiple time point assessment of the salivary biomarkers of SAM system and HPA axis and different parameters of cardiovascular activity after CVT challenge, which is consistent with a distinct activation of the two systems and the HR, SBP, DBP and MAP, showing the same trend changes with α-Amy. Salivary cortisol levels increased during the first 15 minutes following the CVT, likely secondary to the stressful state induced in the study population by the vestibular stimulation.

Although many previous studies have investigated the link between the HPA axis and sympathetic activities in response to different stressful tasks, the complex reciprocal counterbalances have not been entirely defined in terms of the timing and modalities of their activation and interactions [3, 37, 38]. No correlation between α-Amy and cortisol responses and various stress paradigms have been reported [37, 39]. In contrast, we found a significant inverse relationship between the production of α-Amy and cortisol elicited through the air-CVT task. These discordant outcomes may be related to different stress paradigms or to differences in some characteristic of the population sampled: here we enrolled young healthy male subjects with no signs of depression and anxiety [40]. Furthermore, the discrepancy may be related to the different statistical analyses used. In the present study, linear regression analysis was used to predict the trend of each salivary biomarker production in the 60 min following the air CVT and then T tests were used to compare the respective regression coefficients.

Previous studies have differentiated cortisol response, gradually decreasing after repeated TSST, from autonomic reactivity, which was not characterized by habituation [41]. However, we did not examine our study population for repeated stress exposures.

A further limitation of the present study might be that the results cannot be directly generalized because our collection was from a population of young healthy man. Future research will be necessary to confirm the present results in women as well as elderly people. Moreover, our study could not yet clarify another sparse assumption about the possibility that α-Amy could reflect, in time, the balance between sympathetic and parasympathetic activity [2]. However, though future studies will be conclusive, the data presented by us could further support these hypotheses because of the peculiar α-Amy response to the vestibular stimulation, which is aligned with the reduced cardiovascular output.

## Conclusions

Previous studies from our group and others have shown that the measurement of salivary cortisol and α-Amy is becoming more widely accepted for monitoring changes in HPA and SAM activity under stress-related conditions [3,11, 42–46]. Changes in salivary cortisol and α-Amy, as well as their diurnal fluctuations, are thought to have health implications [27, 47–49].

The interactions between stress and vestibular functions have been investigated in animal models and in clinical studies [1, 50] and the possible functional consequences of the consistent SBP, MAP and HR changes evoked by vestibular stimulation have been reported in a healthy population [51]. We cannot exclude that activating component of vestibular apparatus different from those activated in the present study by CVT might elicit different somatic (muscle, skin, cardiovascular output, etc) and neuro-endocrine responses [52]. Further research is needed before we can establish the potential importance of vestibular input to cardiovascular regulation and orthostatic tolerance in humans.

## Supporting information

S1 Dataset Minimal manuscript dataset providing the mean, standard deviation and standard error for the tables and the figures (PDF). (PDF)

S1 Fig Table providing the results of statistical analyses for salivary α-amylase and salivary cortisol in the study population for three time points during a rest day, starting two hours after awake: at 08:00 h, at 12:00 h and 20:00 h. (PDF)

S2 Fig Table providing the results of statistical analyses for salivary α-amylase and salivary cortisol in the study population before and after caloric vestibular test. (PDF)

S3 Fig Table providing the results of statistical analyses for heart rate (HR) and RR interval in the study population before and after caloric vestibular test. (PDF)

S4 Fig Table providing the results of statistical analyses for systolic blood pressure (SBP), diastolic blood pressure (DBP) and mean arterial pressure (MAP) in the study population before and after caloric vestibular test. (PDF)

## References

[1] Saman Y, Bamiou DE, Gleeson M, Dutia MB. Interactions between stress and vestibular compensation—a review. Front Neurol. 2012;3: 1–8. 22866048 10.3389/fneur.2012.00116 PMC3406321

[2] Bosch JA, Veerman EC, de Geus EJ, Proctor GB. α-Amylase as a reliable and convenient measure of sympathetic activity: don't start salivating just yet! Psychoneuroendocrinology. 2011;36 (4): 449–53. doi: 10.1016/j.psyneuen.2010.12.019 21295411 10.1016/j.psyneuen.2010.12.019

[3] Cozma S, Dima-Cozma LC, Ghiciuc CM, Pasquali V, Saponaro A, Patacchioli FR. Salivary cortisol and α-amylase: subclinical indicators of stress as cardiometabolic risk. Braz J Med Biol Res. 2017;50(2): e5577 doi: 10.1590/1414-431X20165577 28177057 10.1590/1414-431X20165577 PMC5390531

[4] Delle Chiaie R, Trabucchi G, Girardi N, Marini I, Pannese R, Vergnani L, et al Group psychoeducation normalizes cortisol awakening response in stabilized bipolar patients under pharmacological maintenance treatment. Psychother Psychosom. 2013;82: 264–66. doi: 10.1159/000348609 23736884 10.1159/000348609

[5] Ghiciuc CM, Cozma-Dima CL, Pasquali V, Renzi P, Simeoni S, Lupusoru CE, et al Awakening responses and diurnal fluctuations of salivary cortisol, DHEA-S and α-amylase in healthy male subjects. Neuroendocrinol Lett. 2011;32: 475–80. 21876512

[6] Ghiciuc CM, Dima Cozma LC, Bercea RM, Lupusoru CE, Mihaescu T, Szalontay A, et al Restoring the salivary cortisol awakening response through nasal continuous positive airway pressure therapy in obstructive sleep apnea. Chronobiol Int. 2013;30: 1024–31. doi: 10.3109/07420528.2013.795155 23859257 10.3109/07420528.2013.795155

[7] Ghiciuc CM, Dima-Cozma LC, Bercea RM, Lupusoru CE, Mihaescu T, Cozma S, et al Imbalance in the diurnal salivary testosterone/cortisol ratio in men with severe obstructive sleep apnea: an observational study. Braz J Otorhinolaryngol. 2016;82(5): 529–35. doi: 10.1016/j.bjorl.2015.09.004 26749455 10.1016/j.bjorl.2015.09.004 PMC9444622

[8] Nater UM, Hoppmann CA, Scott SB. Diurnal profiles of salivary cortisol and alpha-amylase change across the adult lifespan: evidence from repeated daily life assessments. Psychoneuroendocrinology. 2013;38(12): 3167–71. doi: 10.1016/j.psyneuen.2013.09.008 24099860 10.1016/j.psyneuen.2013.09.008 PMC3844069

[9] Patacchioli FR, Ghiciuc CM, Bernardi M, Dima-Cozma LC, Fattorini L, Squeo MR, et al Salivary α-amylase and cortisol after exercise in menopause: influence of long-term HRT. Climacteric. 2015;18(4): 528–35. doi: 10.3109/13697137.2015.1008444 25602168 10.3109/13697137.2015.1008444

[10] Schumacher S, Kirschbaum C, Fydrich T, Ströhle A. Is salivary alpha-amylase an indicator of autonomic nervous system dysregulations in mental disorders? A review of preliminary findings and the interactions with cortisol. Psychoneuroendocrinology. 2013;38: 729–43. doi: 10.1016/j.psyneuen.2013.02.003 23481259 10.1016/j.psyneuen.2013.02.003

[11] Simeoni S, Biselli R, D’Amelio R, Rocca B, Lattanzio S, Mucci L, et al Stress-induced salivary cortisol secretion during hypobaric-hypoxia challenge and in vivo urinary thromboxane production in healthy male subjects. Stress. 2011;14: 282–9. doi: 10.3109/10253890.2010.545458 21434833 10.3109/10253890.2010.545458

[12] Yates BJ, Miller AD. Properties of sympathetic reflexes elicited by natural vestibular stimulation: implications for cardiovascular control. J Neurophysiol. 1994;71: 2087–92. doi: 10.1152/jn.1994.71.6.2087 7931504 10.1152/jn.1994.71.6.2087

[13] Jauregui-Renaud K, Yarrow K, Oliver R, Gresty MA, Bronstein AM. Effects of caloric stimulation on respiratory frequency and heart rate and blood pressure variability. Brain Res Bull. 2010;53: 17–23. 10.1016/s0361-9230(00)00304-x 11033204

[14] Hall KD, Sacks G, Chandramohan D, Chow CC, Wang YC, Gortmaker SL, et al Quantification of the effect of energy imbalance on bodyweight. Lancet 2011;378(9793): 826–37. doi: 10.1016/S0140-6736(11)60812-X 21872751 10.1016/S0140-6736(11)60812-X PMC3880593

[15] Hamilton M. A rating scale for depression. J Neurol Neurosurg Psychiatry. 1960;23: 56–62. 14399272 10.1136/jnnp.23.1.56 PMC495331

[16] Maier W, Buller R, Philipp M, Heuser I. The Hamilton Anxiety Scale: reliability, validity and sensitivity to change in anxiety and depressive disorders. J Affect Disord. 1988;14: 61–8. 2963053 10.1016/0165-0327(88)90072-9

[17] Cohen S, Williamson G. Perceived stress in a probability sample of the United States In Spacapam S. & Oskamp S. (Eds.), The social psychology of health: Claremont Symposium on applied social psychology. Newbury Park. CA: Sage 2008; 31–67.

[18] Kohn PM, Macdonald JE. The survey of recent life experiences: A decontaminated Hassles scale for adults. J Behav Med. 1992;15: 221–36. 1583682 10.1007/BF00848327

[19] British Society of Audiology (BSA). Recommended Procedure: The Caloric Test. Berkshire. UK: British Society of Audiology 2010.

[20] Ganança MM, Bottino MA, Bittar RS, Caovilla HH, Ganança FF. Reference standard to read the air-driven caloric reflex test results. Braz J Otorhinolaryngol. 2009; 75(1): 2 19488553 10.1016/S1808-8694(15)30824-7 PMC9442165

[21] Kasbekar AV, Baguley DM, Knight R, Gomersall P, Parker R, Lloyd SW, et al Heart rate and blood pressure effects during caloric vestibular testing. J Laryngol Otol. 2010;124(6): 616–22. doi: 10.1017/S0022215110000472 20298642 10.1017/S0022215110000472

[22] Duracinsky M, Mosnier I, Bouccara D, Sterkers O, Chassany O. Working Group of the Société Française d'Oto-Rhino-Laryngologie (ORL). Literature review of questionnaires assessing vertigo and dizziness, and their impact on patients' quality of life. Value Health 2007;10(4): 273–84. 17645682 10.1111/j.1524-4733.2007.00182.x

[23] Katz ED, Ruoff BE. Commonly Used Formulas and Calculations, in: Roberts J.R., Hedges J.R. (Eds.), Clinical Procedures in Emergency Medicine. Elsevier Mosby Publishing 2014;1434.

[24] Aiken LS, West SG. Multiple regression: Testing and interpreting interactions. Newbury Park Sage 1991.

[25] Cohen J, Cohen P, West SG, Aiken LS. Applied Multiple Regression/Correlation Analysis for the Behavioral Sciences. 3rd Edition Lawrence, Erlbaum Associated Editors London 2003.

[26] Yumuk V, Tsigos C, Fried M, Schindler K, Busetto L, Micic D, et al Obesity Management Task Force of the European Association for the Study of Obesity. European guidelines for obesity management in adults. Obes Facts. 2015;8: 402–24. doi: 10.1159/000442721 26641646 10.1159/000442721 PMC5644856

[27] Wolf JM, Nicholls E, Chen E. Chronic stress, salivary cortisol, and alpha-amylase in children with asthma and healthy children. Biol Psychol. 2008;78: 20–8. doi: 10.1016/j.biopsycho.2007.12.004 18243483 10.1016/j.biopsycho.2007.12.004

[28] Gordis EB, Granger DA, Susman EJ, Trickett PK. Salivary alpha amylase-cortisol asymmetry in maltreated youth. Horm Behav. 2008;53(1): 96–103. doi: 10.1016/j.yhbeh.2007.09.002 17945232 10.1016/j.yhbeh.2007.09.002 PMC2266091

[29] Dagilas A, Kimiskidis V, Aggelopoulou M, Kapaki E, Fitili C, Libitaki G, et al Changes in blood neurotransmitter and steroid levels during evoked vertigo. Otol Neurotol. 2005;26: 476–80. 15891652 10.1097/01.mao.0000169785.15083.28

[30] Kerman IA, Emanuel BA, Yates BJ. Vestibular stimulation leads to distinct hemodynamic patterning. Am J Physiol Regul Integr Comp Physiol. 2000;279(1): 118–25. 10.1152/ajpregu.2000.279.1.R118 10896872

[31] Biaggioni I, Costa F, Kaufmann H. Vestibular influences on autonomic cardiovascular control in humans. J Vestib Res. 1998;8 (1): 35–41. 9416587

[32] Costa F, Lavin P, Robertson D, Biaggioni I. Effect of neurovestibular stimulation on autonomic regulation. Clin Auton Res. 1995;5(5): 289–93. 8563462 10.1007/BF01818894

[33] Chatterton RT Jr, Vogelsong KM, Lu YC, Ellman AB, Hudgens GA. Salivary alpha-amylase as a measure of endogenous adrenergic activity. Clin Physiol. 1996;16(4): 433–48. 8842578 10.1111/j.1475-097x.1996.tb00731.x

[34] Nater UM, La Marca R, Florin L, Moses A, Langhans W, Koller MM, et al Stress induced changes in human salivary alpha-amylase activity associations with adrenergic activity. Psychoneuroendocrinology. 2006;31(1): 49–58. doi: 10.1016/j.psyneuen.2005.05.010 16002223 10.1016/j.psyneuen.2005.05.010

[35] Kang Y. Psychological stress-induced changes in salivary alpha-amylase and adrenergic activity. Nurs Health Sci. 2010;12(4): 477–84. doi: 10.1111/j.1442-2018.2010.00562.x 21210927 10.1111/j.1442-2018.2010.00562.x

[36] Akizuki K, Yazaki S, Echizenya Y, Ohashi Y. Anaerobic threshold and salivary α-amylase during incremental exercise. J Phys Ther Sci. 2014;26 (7): 1059–63. doi: 10.1589/jpts.26.1059 25140097 10.1589/jpts.26.1059 PMC4135198

[37] Ali N, Pruessner JC.The salivary alpha amylase over cortisol ratio as a marker to assess dysregulations of the stress systems. Physiol Behav. 2012;106 (1): 65–72. doi: 10.1016/j.physbeh.2011.10.003 22019784 10.1016/j.physbeh.2011.10.003

[38] Cortelli P, Lombardi C, Montagna P, Parati G. Baroreflex modulation during sleep and in obstructive sleep apnea syndrome. Auton Neurosci. 2012;169: 7–11. doi: 10.1016/j.autneu.2012.02.005 22465134 10.1016/j.autneu.2012.02.005

[39] Takai N, Yamaguchi M, Aragaki T, Eto K, Uchihashi K, Nishikawa Y. Effect of psychological stress on the salivary cortisol and amylase levels in healthy young adults. Arch Oral Biol. 2004;49: 963–8. doi: 10.1016/j.archoralbio.2004.06.007 15485637 10.1016/j.archoralbio.2004.06.007

[40] Hackney AC, Viru A. Research methodology: endocrinologic measurements in exercise science and sports medicine. J Athl Train. 2008;43: 631–39. doi: 10.4085/1062-6050-43.6.631 19030142 10.4085/1062-6050-43.6.631 PMC2582556

[41] Jönsson P, Wallergård M, Osterberg K, Hansen AM, Johansson G, Karlson B. Cardiovascular and cortisol reactivity and habituation to a virtual reality version of the Trier Social Stress Test: a pilot study. Psychoneuroendocrinology. 2010;35(9): 1397–403. doi: 10.1016/j.psyneuen.2010.04.003 20451329 10.1016/j.psyneuen.2010.04.003

[42] Damian L, Ghiciuc CM, Dima-Cozma LC, Ungureanu MC, Cozma S, Patacchioli FR. et al No definitive evidence for a connection between autoimmune thyroid diseases and stress in women. Neuroendocrinol Lett. 2016;37(3): 155–62. 27618605

[43] Kirschbaum C, Hellhammer DH. Salivary cortisol in psychoneuroendocrine research: recent developments and applications. Psychoneuroendocrinology. 1994;19(4): 313–33. 8047637 10.1016/0306-4530(94)90013-2

[44] Patacchioli FR, Monnazzi P, Scontrini A, Tremante E, Caridi I, Brunetti E, et al Adrenal dysregulation in amyotrophic lateral sclerosis. J Endocrinol Invest. 2003;26(12): 23–5. 10.1007/BF03349149 15055464

[45] Patacchioli FR, Monnazzi P, Simeoni S, De Filippis S, Salvatori E, Coloprisco G, et al Salivary cortisol., dehydroepiandrosterone-sulphate (DHEA-S) and testosterone in women with chronic migraine. J Headache Pain. 2006;7(2): 90–4. doi: 10.1007/s10194-006-0274-6 16575505 10.1007/s10194-006-0274-6 PMC3451699

[46] Pippi R, Patini R, Ghiciuc CM, Sandu RB, Pasquali V, Scaccianoce S, et al Diurnal trajectories of salivary cortisol., salivary α-amylase and psychological profiles in oral lichen planus patients. J Biol Regul Homeost Agents. 2014;28(1): 147–54. 24750801

[47] Ahn RS, Lee YJ, Choi JY, Kwon HB, Chun SI. Salivary cortisol and DHEA levels in the Korean population: age-related differences, diurnal rhythm, and correlations with serum levels. Yonsei Med Journal. 2007;48: 379–88. 17594144 10.3349/ymj.2007.48.3.379 PMC2628086

[48] Chida Y, Steptoe A. Cortisol awakening response and psychosocial factors: a systematic review and meta-analysis. Biol Psychol. 2009;80: 265–78. doi: 10.1016/j.biopsycho.2008.10.004 19022335 10.1016/j.biopsycho.2008.10.004

[49] Patacchioli FR, Tabarrini A, Ghiciuc CM, Dima-Cozma LC, Prete A, Bianchini C, et al Salivary biomarkers of obstructive sleep apnea syndrome in children. Pediatr Pulmonol. 2014;49(11): 1145–52. doi: 10.1002/ppul.22972 24474530 10.1002/ppul.22972

[50] Archana R, Sai Sailesh Kumar, Mukkadan JK. Effect of vestibular stimulation on stress and cardiovascular parameters in healthy college students. Biomedical Research 2016;27(3): 985–990.

[51] Hallgren E, Migeotte PF, Kornilova L, Delière Q, Fransen E, Glukhikh D, et al Dysfunctional vestibular system causes a blood pressure drop in astronauts returning from space. Sci Rep. 2015;5: 17627 doi: 10.1038/srep17627 26671177 10.1038/srep17627 PMC4680856

[52] Hammam E and Macefield VG. Vestibular modulation of sympathetic nerve activity to muscle and skin in humans. Front Neurol. 2017;8: 334 doi: 10.3389/fneur.2017.00334 28798718 10.3389/fneur.2017.00334 PMC5526846

