# Supplementary material for: Distinct activation of the sympathetic adreno-medullar system and hypothalamus pituitary adrenal axis following the caloric vestibular test in healthy subjects
Source: PLoS One. 2018 Mar 6;13(3):e0193963. doi: 10.1371/journal.pone.0193963 (PMC5839583; doi:10.1371/journal.pone.0193963)
Supplement: S1 Dataset — (PDF) [file pone.0193963.s001.pdf]

	CALORIC TEST - Socio-demographic characteristics													
CODE VER	Age	Educational level	Education (years)	Marital status	Physical activity (min/day)	hours of sleep (h)	Weight (kg)	Heigh (cm)	BMI	abd circum (cm)	hip circum (cm)	abd /hip ratio	SBP (mm Hg)	DBP (mm Hg)	HR (b/ min)
AVERAGE for 1-48	24,7	3,0	16,7	1,0	64,2	7,3	75,6	178,9	23,6	86,1	102,5	0,8	121	77	72
±	±	±	±	±	±	±	±	±	±	±	±	±	±	±	±
STD	2,2	0,0	1,6	0,2	35,8	1,0	11,2	6,9	3,0	7,2	5,8	0,0	12	9	9
SE	0,3	0,0	0,2	0,0	5,5	0,1	1,6	1,0	0,4	1,0	0,8	0,0	1,7	1,2	1,3

CALORIC TEST - BLOOD PRESSURE AND HEART RATE

CODE VER	Nistagmus		dizziness - Lickert scale 1-10	Systolic BP (mmHg)								
				Before stimulation 1	after nistagmus							
	begins after (sec)	duration (sec)			1' after 3	4' after 4	7' after 5	10' after 6	15' after 7	30' after 8	45' after 9	60' after 10
AVERAGE for 1-48	41	127	8	122,2	125,2	120,7	119,4	116,9	115,6	118,9	118,9	119,4
±	±	±	±	±	±	±	±	±	±	±	±	±
STD	12	28	2	12	13	10	11	11	11	8	8	9
SE	2,3	5,2	0,4	2,2	2,5	1,9	2,0	2,0	2,0	1,5	1,4	1,6

CODE VER	Dyastolic BP (mmHg)								
	Before stimulation 1	after nistagmus							
		1' after 3	4' after 4	7' after 5	10' after 6	15' after 7	30' after 8	45' after 9	60' after 10
AVERAGE for 1-48	77,8	78,6	76,3	75,2	76,1	76,0	79,1	78,6	79,9
±	±	±	±	±	±	±	±	±	±
STD	9	7	8	9	9	7	8	8	8
SE	1,7	1,3	1,5	1,7	1,6	1,4	1,6	1,5	1,5

CODE VER	Mean arterial pressure (MAP) (mmHg)								
	Before stimulation 1	after nistagmus							
		1' after 3	4' after 4	7' after 5	10' after 6	15' after 7	30' after 8	45' after 9	60' after 10
AVERAGE for 1-48	92,6	94,2	91,1	89,9	89,7	89,2	92,4	92,0	93,0
±	±	±	±	±	±	±	±	±	±
STD	9	9	8	9	9	8	7	7	7
SE	1,7	1,6	1,5	1,7	1,6	1,4	1,3	1,2	1,2

HOLTER ECG

CODE VER	HR (b/mi	HR 1 mi	HR 4 mi	HR 7 mi	HR 10 m	HR 15 m	HR 30 m	HR 45 m	HR 60 min
AVERAGE for 1-48	70,8	79,5	70,6	64,3	62,9	59,5	67,0	67,6	70,7
±	±	±	±	±	±	±	±	±	±
STD	9,5	9,0	10,7	11,3	9,6	9,1	9,0	9,4	5,3
SE	1,4	1,3	1,5	1,6	1,4	1,3	1,3	1,4	0,8

HOLTER ECG

RR (ms)	RR 1 mi	RR 4 mi	RR 7 mi	RR 10 mi	RR 15 mi	RR 30 mi	RR 45 mi	RR 60 min
862,0	763,0	869,0	961,0	973,0	1034,0	914,0	905,0	854,0
±	±	±	±	±	±	±	±	±
108,0	88,0	127,0	170,0	143,0	185,0	135,0	136,0	61,0
16,0	13,0	18,0	25,0	21,0	27,0	20,0	20,0	9,0

SALIVARY CORTISOL CALORIC TEST (ng/ml)									
CODE VERTIGO	before 1	after stimulation							
		1' after 3	4' after 4	7' after 5	10' after 6	15' after 7	30' after 8	45' after 9	60' after 10
AVERAGE for 1-48	3,2	3,6	3,8	4,7	4,6	4,8	3,5	3,4	3,3
±	±	±	±	±	±	±	±	±	±
STD	1,8	2,2	2,1	3,1	3,4	3,6	3,1	3,6	3,5
SE	0,3	0,3	0,3	0,5	0,5	0,5	0,4	0,5	0,5

SALIVARY α-AMYLASE CALORIC TEST (U/ml)									
CODE VERTIGO	before stimulation 1	after stimulation							
		1' after 3	4' after 4	7' after 5	10' after 6	15' after 7	30' after 8	45' after 9	60' after 10
AVERAGE for 1-48	33,4	25,5	24,7	24,2	21,3	24,8	37,7	38,7	34,2
±	±	±	±	±	±	±	±	±	±
STD	9,1	14,5	16,4	15,1	14,6	17,2	21,9	14,6	21,3
SE	1,3	2,1	2,4	2,2	2,1	2,5	3,2	2,1	3,1

	CALORIC TEST<br>SALIVARY CORTISOL AWAKE (ng/ml)				
CODE VER	COR 08:00 ortost 1	COR 12:00 ortost 2	COR 20:00 ortost 3	COR AUC	diurnal fluctuation (M/E)*100 ortost
AVERAGE for 1-48	5,6	3,0	1,7	35,9	367,9
±	±	±	±	±	±
STD	2,3	1,3	0,7	13,1	189,0
SE	0,3	0,2	0,1	1,9	27,3

	CALORIC TEST<br>SALIVARY α-AMYLASE AWAKE (U/ml)				
CODE VER	AMY 08:00 ortost 1	AMY 12:00 ortost 2	AMY 20:00 ortost 3	AMY AUC	diurnal fluctuation (M/E)*100 ortost
AVERAGE for 1-48	22,2	26,5	34,3	340,2	72
±	±	±	±	±	±
STD	11,1	13,0	14,9	133,9	49
SE	1,6	1,9	2,1	19,3	7,1

CALORIC TEST	PSYCHOMETRIC TESTS - ENROLLMENT		PSYCHOMETRIC TESTS - ENROLLMENT		PSYCHOMETRIC TESTS AFTER CALORIC TEST	
CODE VER	HDS	HAS	Hassles	PSS (Cohen-Williamson)	Hassles	PSS (Cohen-Williamson)
AVERAGE for 1-29	3,5	3,0	73	29	87	35
±	±	±	±	±	±	±
STD	2,1	2,1	7	3	12	4
SE	0,3	0,3	1,0	0,4	1,8	0,6
