# Supplementary material for: Distinct activation of the sympathetic adreno-medullar system and hypothalamus pituitary adrenal axis following the caloric vestibular test in healthy subjects
Source: PLoS One. 2018 Mar 6;13(3):e0193963. doi: 10.1371/journal.pone.0193963 (PMC5839583; doi:10.1371/journal.pone.0193963)
Supplement: S1 Fig — (PDF) [file pone.0193963.s002.pdf]

TABLE Diurnal trajectories of Salivary α-Amylase (U/ml) and Salivary Cortisol (ng/ml) on rest day (n=48)

	08:00 h	12:00 h	20:00 h
Salivary α-amylase (U/ml)	22.2 ± 1.6 (11.1)	26.5 ±1.9 (13.0) *	34.3 ±2.1 (14.9) *
Salivary cortisol (ng/ml)	5.6 ±0.3 (2.3)	3.0 ±0.2 (1.4) *	1.7 ±0.1 (0.7) *

Data are shown as mean values ± SE (SD).

Statistical Analysis One Way Repeated Measures Analysis of Variance:
Salivary α-amylase: $F_{(2, 143)}$= 19.809; p<0.001;
Post hoc Tuckey test for multiple comparison: *: p<0.001 versus 08:00.

Salivary cortisol: $F_{(2, 143)}$= 135.843; p<0.001;
Post hoc Tuckey test for multiple comparison: *: p<0.001 versus 08:00.
