# Supplementary material for: Distinct activation of the sympathetic adreno-medullar system and hypothalamus pituitary adrenal axis following the caloric vestibular test in healthy subjects
Source: PLoS One. 2018 Mar 6;13(3):e0193963. doi: 10.1371/journal.pone.0193963 (PMC5839583; doi:10.1371/journal.pone.0193963)
Supplement: S2 Fig — (PDF) [file pone.0193963.s003.pdf]

TABLE Salivary α-Amylase (U/ml) in the Study Population – caloric vestibular test (n=48)

	before CVT	caloric vestibular test (CVT)							
		1' after	4' after	7' after	10' after	15' after	30' after	45' after	60' after
Salivary α-amylase (U/ml) (n=48)	33.4 ± 1.3 (9.1)	25.5 ±2.1 (14.5) *	24.7 ±2.4 (16.4) **	24.2 ±2.2 (15.1) **	21.3 ± 2.1 (14.6) **	24.8 ±2.5 (17.2) **	37.7 ±3.2 (21.9)	38.7 ±2.1 (14.6)	34.2 ±3.1 (21.3)

Data are shown as mean values ± SE (SD).
Statistical Analysis One Way Repeated Measures Analysis of Variance:
Salivary α-amylase: for factor Time $F_{(8, 431)}$= 13.584; $p<0.001$;
Post hoc Tukey Test for multiple comparison: *: $p<0.01$, **: $p<0.001$ versus before CVT.

TABLE Salivary Cortisol (ng/ml) in the Study Population – caloric vestibular test (n=48)

	before CVT	caloric vestibular test (CVT)							
		1' after	4' after	7' after	10' after	15' after	30' after	45' after	60' after
Salivary cortisol (ng/ml) (n=48)	3.2 ±0.3 (1.8)	3.6 ±0.3 (2.2)	3.8 ±0.3 (2.1)	4.7 ±0.5 (3.1) **	4.6 ±0.5 (3.4) *	4.8 ±0.5 (3.4) **	3.5 ±0.4 (3.1)	3.4 ±0.5 (3.6)	3.3 ±0.5 (3.5)

Data are shown as mean values ± SE (SD).

Statistical Analysis One Way Repeated Measures Analysis of Variance:
Salivary cortisol: for factor Time $F_{(8, 431)}$= 4.432; $p<0.001$;
Post hoc Tukey test for multiple comparison: *: $p<0.01$, **: $p<0.001$ versus before CVT.
