# Supplementary material for: Distinct activation of the sympathetic adreno-medullar system and hypothalamus pituitary adrenal axis following the caloric vestibular test in healthy subjects
Source: PLoS One. 2018 Mar 6;13(3):e0193963. doi: 10.1371/journal.pone.0193963 (PMC5839583; doi:10.1371/journal.pone.0193963)
Supplement: S3 Fig — (PDF) [file pone.0193963.s004.pdf]

HR

TABLE Cardiovascular parameters in the Study Population – caloric test (n=48)

	before caloric test	CVT							
		1' after	4' after	7' after	10' after	15' after	30' after	45' after	60' after
Heart rate (beats/min)	71 ± 1.4 (9.5)	79 ± 1.3 (9.0) **	71 ± 1.5 (10.7)	64 ± 1.6 (11.3) *	63 ± 1.4 (9.6) **	60 ± 1.3 (9.1) **	67 ± 1.3 (9.0)	68 ± 1.4 (9.4)	71 ± 0.8 (5.8)

Data are shown as mean values ± SE (SD).

Statistical Analysis One Way Repeated Measures Analysis of Variance:
HR: for factor Time $F_{(8, 431)}$= 27.027; $p<0.001$;
Post hoc Tukey for multiple comparison: *: $p<0.01$, **: $p<0.001$ versus before CVT.

RR interval

TABLE Cardiovascular parameters in the Study Population – caloric test (n=48)

	before caloric test	CVT							
		1' after	4' after	7' after	10' after	15' after	30' after	45' after	60' after
RR interval (ms)	862 ± 16 (108)	763 ± 13 (88) **	869 ± 18 (127)	961 ± 25 (170) **	973 ± 21 (143) **	1034 ± 27 (185) **	914 ± 20 (135)	905 ± 20 (136)	854 ± 9 (61)

Data are shown as mean values ± SE (SD).

Statistical Analysis One Way Repeated Measures Analysis of Variance:
RR interval: for factor Time $F_{(8, 431)}$= 26.262; $p<0.001$;
Post hoc Tukey Test for multiple comparison: **: $p<0.001$ versus before CVT.
