# Supplementary material for: Distinct activation of the sympathetic adreno-medullar system and hypothalamus pituitary adrenal axis following the caloric vestibular test in healthy subjects
Source: PLoS One. 2018 Mar 6;13(3):e0193963. doi: 10.1371/journal.pone.0193963 (PMC5839583; doi:10.1371/journal.pone.0193963)
Supplement: S4 Fig — (PDF) [file pone.0193963.s005.pdf]

TABLE Cardiovascular parameters - blood pressure (mmHg) in the Study Population – caloric test (n=48)

	before CVT	caloric vestibular test (CVT)							
		1' after	4' after	7' after	10' after	15' after	30' after	45' after	60' after
SBP (n=48)	122 ± 2.2 (12)	125 ± 2.5 (13)	121 ± 1.9 (10)	119 ± 2.0 (11)	117 ± 2.0 (11) ***	116 ± 2.0 (11) ***	119 ± 1.5 (8)	119 ± 1.4 (8)	119 ± 1.6 (9)

Data are shown as mean values ± SE (SD).

Statistical Analysis One Way Repeated Measures Analysis of Variance:
SBP: for factor Time $F_{(8, 431)}$= 11.179; p<0.001;
Post hoc Tuckey Test for multiple comparison: ***: p<0.001 versus before CVT.

TABLE Cardiovascular parameters - blood pressure (mmHg) in the Study Population – caloric test (n=48)

	before CVT	caloric vestibular test (CVT)							
		1' after	4' after	7' after	10' after	15' after	30' after	45' after	60' after
DBP (n=48)	78 ± 1.7 (9)	79 ± 1.3 (7)	76 ± 1.5 (8)	75 ± 1.7 (9)	76 ± 1.6 (9)	76 ± 1.4 (7)	79 ± 1.6 (8)	79 ± 1.8 (8)	80 ± 1.5 (8)

Data are shown as mean values ± SE (SD).

Statistical Analysis One Way Repeated Measures Analysis of Variance:
DBP: for factor Time $F_{(8, 431)}$= 5.370; p<0.001;
Post hoc Tukey Test for multiple comparison: not significant versus before CVT.

TABLE Cardiovascular parameters - blood pressure (mmHg) in the Study Population – caloric test (n=48)

	before CVT	caloric vestibular test (CVT)							
		1' after	4' after	7' after	10' after	15' after	30' after	45' after	60' after
MAP (n=48)	93 ± 1.7 (9)	94 ± 1.6 (9)	91 ± 1.5 (8)	90 ± 1.7 (9) *	90 ± 1.6 (9) *	89 ± 1.4 (8) **	92 ± 1.3 (7)	92 ± 1.2 (7)	93 ± 1.2 (7)

Data are shown as mean values ± SE (SD). MAP = [(2 x diastolic)+systolic] / 3

Statistical Analysis One Way Repeated Measures Analysis of Variance:
MAP: for factor Time $F_{(8, 431)}$= 8.069; p<0.001;
Post hoc Tuckey Test for multiple comparison: *,**: p<0.05, p<0.01 versus before CVT.
